# Historical Reminiscences

**Published:** 1886-02

**Authors:** C. S. Chittenden

**Affiliations:** Hamilton, Ont.


### HISTORICAL REMINISCENCES.

BY C. S. CHITTENDEN, D. D. S., L. D. S., HAMILTON, ONT.

READ BEFORE A UNION MEETING OF THE SEVENTH AND EIGHTH DISTRICT DENTAL SOCIETIES OF THE STATE OF NEW YORK, HELD IN BUFFALO, OCT. 27 AND 28, 1885.

Fifty years ago, as I was walking down St. Paul street, Burlington, Vermont, with my father, I read, on a door-plate, " O. H. Saxton, Dentist." I asked what the word " dentist " meant, when my father replied: " It means a man who treats people's teeth. It is a very lucrative business, I understand," and this led me to inquire further, as to the meaning of the word lucrative.

I was then ten years of age, and had never heard of a dentist. Two years later, in 1837, I came west to visit my older brothers, one of whom, Dr. Nelson Chittenden, then of Nunda, N. Y., I found to be a dentist. I saw him cut up calves' teeth, for insertion in the mouths of his patients. I well remember how ghastly the calves' jaws looked to me. He also carved teeth from the hippopotamus tusk, and fastened them in the mouth with gold wires passing through the artificial teeth and around the natural ones, and thus they were tolerably well fixed in the mouth. I remember his showing my father some mineral teeth, which he thought might possibly supplant those made from the teeth of animals. Nine more years passed, when I found myself again with my brother. I was then twenty-one years old, and was starting out in life. My brother, who was nineteen years my senior, advised me to study dentistry. I accepted his advice, and commenced.

I said "study" dentistry, but there was really so little to study that, practically, it meant watching him in the operating-room and the laboratory, and receiving his oral instructions. Thirty-nine years have passed since then, during which time I have heard many lectures, read many volumes, attended many dental meetings, where I have heard the best dentists in the country describe their operations, but I have seen or heard of few who performed in the mouth or in the laboratory more conscientiously or successfully than he. If he were not certain of success he refused to act at all. The only book he had for me to study was Harris' Practice, then about one-fourth its present size. He was anxious to keep abreast of the times, but there was very little to keep abreast of, as we look at the profession now. He had a set of forceps made, I think, by Bushnell, of Rochester, which were certainly strong, and able to resist any amount of force. I once asked him why he never used these forceps for extracting, when he told me that unless the teeth were very strong and solid the forceps would crush them. The turnkey was his favorite instrument, and he certainly could use it most effectually. It mattered not which, he could extract with it any tooth that could be extracted at all.

I was taught never to use amalgam, and did not till I had been in practice for quite a number of years. Gold and tin foils were the only materials employed at that time, so far as I know, for filling teeth. These were rolled into ropes, one end turned on itself, forced into a retaining point and fixed. Then another loop or turn on itself was forced down beside the first, and so on till the cavity was filled, when the whole surface would be condensed, smoothed and burnished. I doubt if I could make a good filling in that way now, but I did then, and many of the fillings of those days preserved the teeth as effectually as our more scientifically inserted ones do now. A half-dozen cherry headed drills, as many hatchet-shaped excavators, three or four incisor and as many molar files, with a half-dozen queer-shaped filling instruments, completed the outfit for the operative department. No napkins were needed, as while it was considered rather desirable, it was not thought necessary to keep a cavity dry while filling. I remember hearing Dr. Westcott, of Syracuse, say that he had frequently seen the late Dr. C. A. Harris fill teeth without the slightest attempt to exclude the saliva.

In the mechanical department the first requisite was a pair of rollers, or, as our English friends call them, "flatters," three or four impression cups (for wax only), with a plate-punch, one or two pairs of pliers for holding gold while filing, a pair of scissors, with lead and tin for swages, and the office was complete. A lathe was a superfluity in those days. A small grinding-stone (such as is used for sharpening knives) sufficed for grinding teeth, the polishing of plates being done by hand, with pencils and sticks dipped in oil and emery. The fee for an upper or lower set of teeth on gold was fifty dollars; on silver, half that sum. So far as I can remember, the above were the fees among all the dentists for mechanical work. For filling with gold, seventy-five cents; tin or amalgam, thirty-seven and a half cents. I have no doubt that much higher fees were charged in large towns, but I am not certain.

There was but one dental college in the world at that time, and but one dental paper, viz., *The American Journal of Dental Science.* This was published regularly. Dr. E. Parmly published a paper which, so far as I can remember, was issued "from occasionally to semi-occasionally." I think it was called *The Dental Intelligencer.* I have before me the first number of the *New Dental Recorder*, dated Sept 1st, 1846. There is no name attached to it as editor or publisher, but I think Dr. C. C. Allen acted as the former, for I find his name as such in subsequent numbers. It can easily be imagined how eagerly I devoured each number as it appeared, and with what delight I read "Maury's Dental Surgery," and another by Paul B. Goddard, which I had obtained before the end of 1847. When I look over these old journals and see such names as Geo. E. Hawes, J. F. B. Flagg, Amos Westcott, W. H. Dwinelle, John Burdell, E. Parmly, E. Townsend, James Taylor, C. A. Harris, J. D. White, John Allen, E. J. Dunning, Robt. Arthur, S. P. Miller, P. B. Goddard, T. L. Buckingham, and a host of others, I feel that I am in the sere and yellow leaf. All these men did good work, not only for their patients, but for us who are alive and remain. "There were giants in those days."

On the first of September, 1847, *The Dental News Letter*, published by Jones & White, appeared. It was a newsy, spicy journal, and reads well even now. It was continued through twelve volumes, when it was succeeded by *The Dental Cosmos*, in 1859. In 1847, our old friends, Drs. Taft and Watt, commenced *The Dental Register* (I am not quite certain that Messrs. Taft and Watt were the original editors).* This journal has always done most useful work. I am delighted to know that both of these gentlemen are still in the flesh, but sorry to hear that Dr. Watt is disabled from active duty.

Many other journals sprang up and ran a short course, but as they were mostly published in the interest of some man or clique who had a whimsey to air, they lived through but few numbers.

In returning to my own personal recollections, I may say that I returned to Vermont and entered the office of Dr. Marcellus Newton, of Montpelier, in the autumn of 1847. He was one of the best manipulators of soft foil that I have ever met. I found him to be a kind, patient gentleman, who did all in his power to advance me in the knowledge of my profession, and in 1848 offered me a partnership, which I accepted, but before articles were drawn I received so great an injury to one of my feet that I was disabled from attending to business for a good many months. In 1849 I was urged by a friend to settle in Hamilton, Ont., where I have remained ever since.

I at once took possession of the office which had been occupied by the late Dr. Alvan Blakesley, of Utica, and more recently by Dr. E. S. Holmes, now of Grand Rapids, Michigan. Each of these gentlemen left a good name behind him, and for years I met evidences of their skill as dentists. Dr. Blakesley was a most excellent operative dentist, with very marked individuality about him. On commencing practice in Hamilton, I met the first amalgam fillings I had ever seen. Frequently persons wearing eight or ten amalgam fillings, all as black as could well be, would call on me for more of the same kind, but I had been too scrupulously trained to avoid all compounds containing mercury, as rank poison, to be

---

* The first number of *The Dental Register* was issued October, 1847, as a quarterly journal, edited by James Taylor, of Cincinnati, and B. B. Brown, of St. Louis.—EDITOR.

willing to use it at all, and years passed before I could overcome my scruples. My patients were largely from the old country, where gold was, at that time, almost never employed for tooth filling; where "amalgam was the best of anything," etc., etc.; and so—well—you know it is said that—

> "Vice is a monster of so frightful mien
> As to be hated needs but to be seen;
> Yet seen too oft, familiar with her face,
> We first endure, then pity, then embrace."

—And so I fell. I have used it ever since, but conscience gives me an occasional twinge yet. I hope my son will live to see something better substituted for it.

In 1852, a Mr. Trendelmberg, of Williamsburg, introduced a preparation consisting of equal parts of calcined bone, sulphur and feldspar, pulverized, for filling teeth. The sulphur was melted and the other ingredients stirred in till the compound was sufficiently consistent. It was called "Stone Filling." As might be expected, it died in infancy.

In 1851, Dr. John Allen promulgated his system or process of mounting teeth on a platinum base. Dr. Wm. M. Hunter invented a somewhat similar process, both having continuous gums, and a war of words was carried on for a long time. In the end Allen succeeded, and no one appears to have heard of Hunter's process for years.

In the same year Dr. S. P. Hullihen, of Wheeling, Va., set forth a new system for treating exposed pulps, which he and somebody else named "Rhizodontryphy-Neurhamaxis," and yet the world revolved much as it did before.

In common with many others I "Rhizodontryphied" a great many nerves. A few did well, but the operation was so unsatisfactory that it was soon dropped. It appears that a Mr. S. P. Miller, of Worcester, Mass., discovered or invented the identical operation, which the Boston people called "Neurhamaxis," and the eastern people "Neurhamaxised" nerves for a year or two, during which the Hullihen partisans fired "Rhizodontryphy," and the Miller partisans let fly "Neurhamaxis" at each other, till it was most alarming. Both are at rest now.

In April, 1853, Mr. Alfred J. Watts, of Utica, N. Y., obtained a patent for the manufacture of sponge gold. This was the first move in the direction of contour filling. For some time Mr. Watts was not successful in producing gold that would save teeth, and complaints were made of a blue line about the margins of the cavities filled with it, which would increase till the filling could be proved to be defective. The profession ought to raise a monument to Mr. Watts, as it was from this preparation of gold that the first adhesive foil was made.

Dr. Spooner had, some years before this, introduced the idea of destroying pulps with arsenic, and in my early dental days many dentists were in the habit of devitalizing the pulp on one day and filling it on the next, without attempting to remove the pulp at all. As a matter of course, there was trouble in most cases, so that, too, soon lost caste.

About 1854, some one suggested the idea of retaining partial sets of teeth by atmospheric pressure. I cannot remember who first proposed this method of insertion, but it met with a great deal of opposition at the outset, and did not come into general use for some years.

In 1855, Mr. J. R. Quinton, of London, Eng., introduced congelation as a local anæsthetic. Like hundreds, I invested my hard-earned dollars in two or three different forms of apparatus for producing local anæsthesia, but it was a decided failure.

In 1856, rubber was brought out as a base for artificial teeth. Probably no one appliance pertaining to dentistry has ever been received and adopted by the profession so universally as this one. It is *the base* which is found where artificial teeth are inserted. In the same year Blandy's cheoplastic method of mounting artificial teeth was given to the public. It had its advantages for lower sets, but rubber soon pushed it to one side.

In the early part of 1857, Dr. Harvey Burdell was found dead in his office, having been murdered by some unknown person during the previous night. His taking off created a great sensation at the time, but no definite solution of the cause or manner has ever been made.

In the same year Dr. Robt. Arthur informed the profession that he had discovered a simple method of keeping proximate cavities dry while operating, which was to cut off a piece of rubber tubing, such as is used in regulating teeth, and slip it over the crown, and up or down on the neck of the tooth to be filled, thus forcing the gums away. It was a move in the right direction. It was in this same year that Slayton's colored gutta-percha base for artificial teeth was brought out. Of all the innumerable humbugs that have been imposed on the profession this was about the biggest.

I ought to have stated that the American Dental Convention was formed, or rather organized, at Philadelphia during the 2d, 3d and 4th days of August, 1855. I felt a little curiosity to look over the list of names of those who attended that convention, and was surprised to find only one name from Western New York, viz., Dr. Fenn, of Rochester.

This meeting had been called by a committee of members of the profession, which met at Philadelphia during the previous year. A constitution had been formed, and everything so carefully prepared by the committee that the convention was born and ready for action on the day of its birth.

In 1856, the convention met in Hope Chapel, New York, on the 6th of August, and continued in session through the 7th and 8th. I find no names from Western New York, except L. D. Walter, of Lockport, B. R. McGregor, of Rochester, and S. B. Palmer, of Tully.

In 1857, the convention met in Boston, at which meeting no Western New York men were present, except our old friend, Dr. Palmer.

The year 1859 is the most memorable one in the annals of dental science. On the 3d of August, of that year, delegates from the Cincinnati Dental Association, Ohio College of Dental Surgery, Mississippi Valley Association, Ohio Dental College Association, Pennsylvania College of Dental Surgery, Western Dental Association, Penn. Association of Dental Surgeons, Indiana State Dental Association, St. Louis Dental Association, and Pittsburg Dental Association met at Niagara Falls, "to determine upon the expediency of forming a National Dental Association upon a representative basis," and, although a meeting of the dentists of New York city, held during the previous month, passed resolutions protesting against the formation of such an association, declaring that the American Dental Convention was all that was required, the delegates proceeded to, and did, form the American Dental Association. Meetings have been held annually since, with the exception of the first year of the late war, 1861. The work performed by the Association has been most extensive and valuable, and is as familiar to you as to me. The Dental Convention, too, proved most useful, but in another direction.

In January, 1867, Dr. B. W. Day, of Kingston, Ont., called a meeting of the dentists of the Province, at Toronto, for the purpose of asking the legislature to incorporate the profession. As only seven responded to the call, those present formed themselves into a dental society. A committee was appointed to draft a bill, which was to be laid before a meeting of the dentists of the Province, to be held at Cobourg in the following July. The meeting at Cobourg was well attended; the draft of the bill was fully considered, and eight persons were recommended to Parliament to represent the profession on the Board of Examiners. Let it be remembered that this was the first attempt at dental legislation.* As it had to be, in the nature of things, a popular measure with the profession in order to get it passed at all, the wonder is that it is as good a bill as it is. It is awkward in many of its provisions, and hard to be understood in others, still I think it is the best dental act in existence. Petitions poured into the legislature from many quarters against the passage of the act. The promoters were at times greatly discouraged, but, as "All's well that ends well," on the last night of the session the bill was read a third time and passed. It is not necessary to add more on the subject than to say that the act has been very successfully carried out from the first, and that there are now very few practicing illegally in the Province. Since the passage of the Ontario act, the Province of Quebec, England, several of the Continental States of Europe, and most of the different States of the American Union have legislated upon the subject of dental practice; but whether the profession stands in a better position now than it did previous to any legislation, is a matter of question which time alone can decide.

I cannot close this paper without speaking of the different manners of dentists in my early dental life, and now. Then, if you called on a brother practitioner he would treat you as an interloper, and if you proposed to speak on matters dental, he would tell you (as I have been told) that "no information can be obtained from me without pay." Now, we meet like brothers, and consult

* The State of Alabama had upon her statute books a very stringent dental law, which antedates that of Canada by nearly ten years.—EDITOR.

on the best methods of procedure. The dentists of to-day can well quote Mark Twain's saying—"the amount that the ancients didn't know is voluminous"—and not be far astray, and yet they did a great deal of good work, when the appliances they possessed are taken into consideration. Let me speak of a few of those which we consider perfectly indispensable now, but which were unknown in my early life: The operating chair, with all its conveniences; the lathe; napkins; duct compressors; the hand, the automatic, and the electric mallets; the dental engine in its various forms; the rubber dam; the different adhesive forms of gold; and many, many other appliances of minor yet of great importance to the dental operator. The profession at large owes a debt to the old Western New York Society, as a great number of these appliances are the inventions of some of its members. From one, dental colleges have become legions, almost. Dental societies have sprung up all over the land. Dental journals, from one or two small quarterlies then, to numerous large, well-edited periodicals of the present. Then, the dentist was scorned by the physicians; now, he is treated like a brother, and there seems little doubt that the dentist of the near future will be an acknowledged specialist of the medical profession.

